# Supplementary figures and images for: Probing neural networks for dynamic switches of communication pathways
Source: PLoS Comput Biol. 2019 Dec 16;15(12):e1007551. doi: 10.1371/journal.pcbi.1007551 (PMC6936858; doi:10.1371/journal.pcbi.1007551)

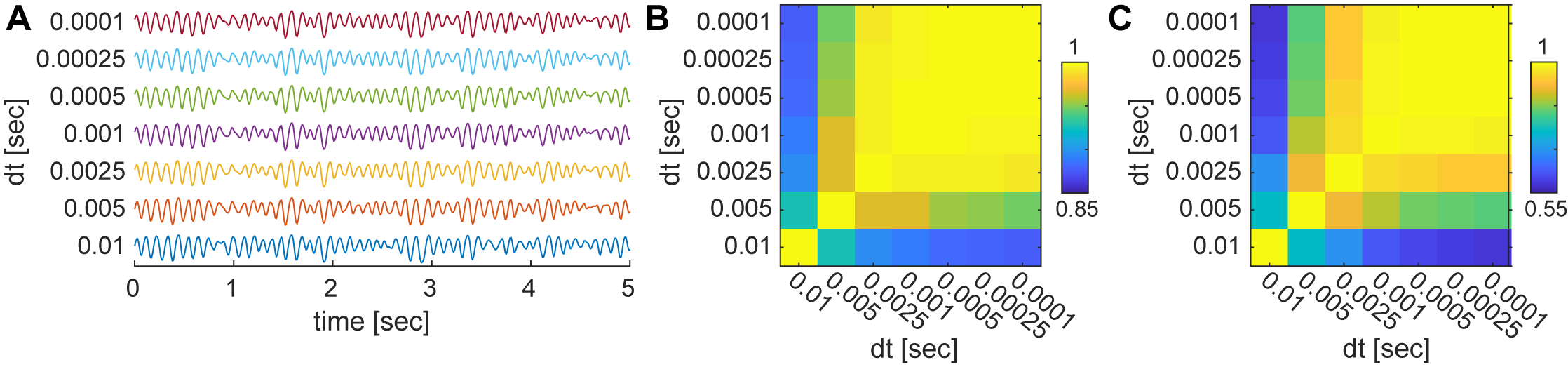

Supplement: S1 Fig — (A) comparison of the timeseries for different step sizes of the Runge-Kutta integration method. All time series are of the same node that is embedded in the connectome network of 33 nodes. (B) The correlation matrix between the timeseries using different simulation step sizes. (C) The correlation between functional connectivity matrices obtained using different simulation step sizes. (TIF) [file pcbi.1007551.s001.tif]

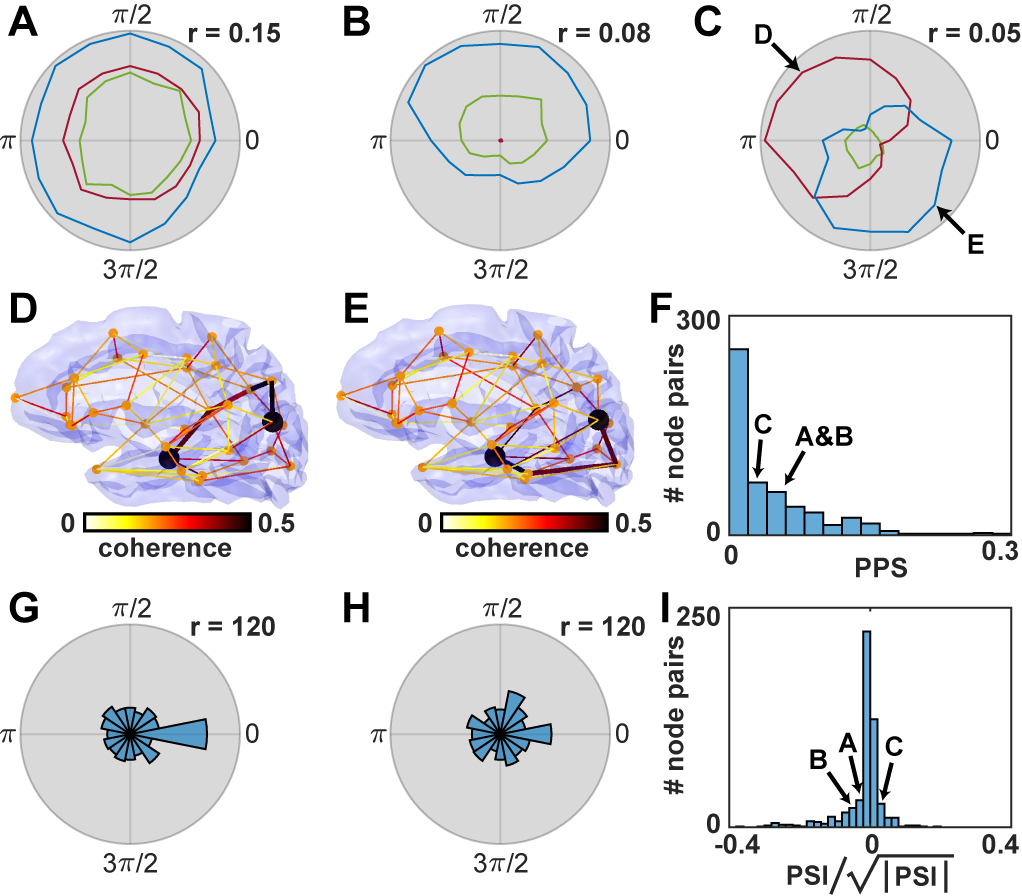

Supplement: S2 Fig — The panels correspond to Fig 7 in the main text. Please find the detailed panel descriptions there. (TIF) [file pcbi.1007551.s002.tif]

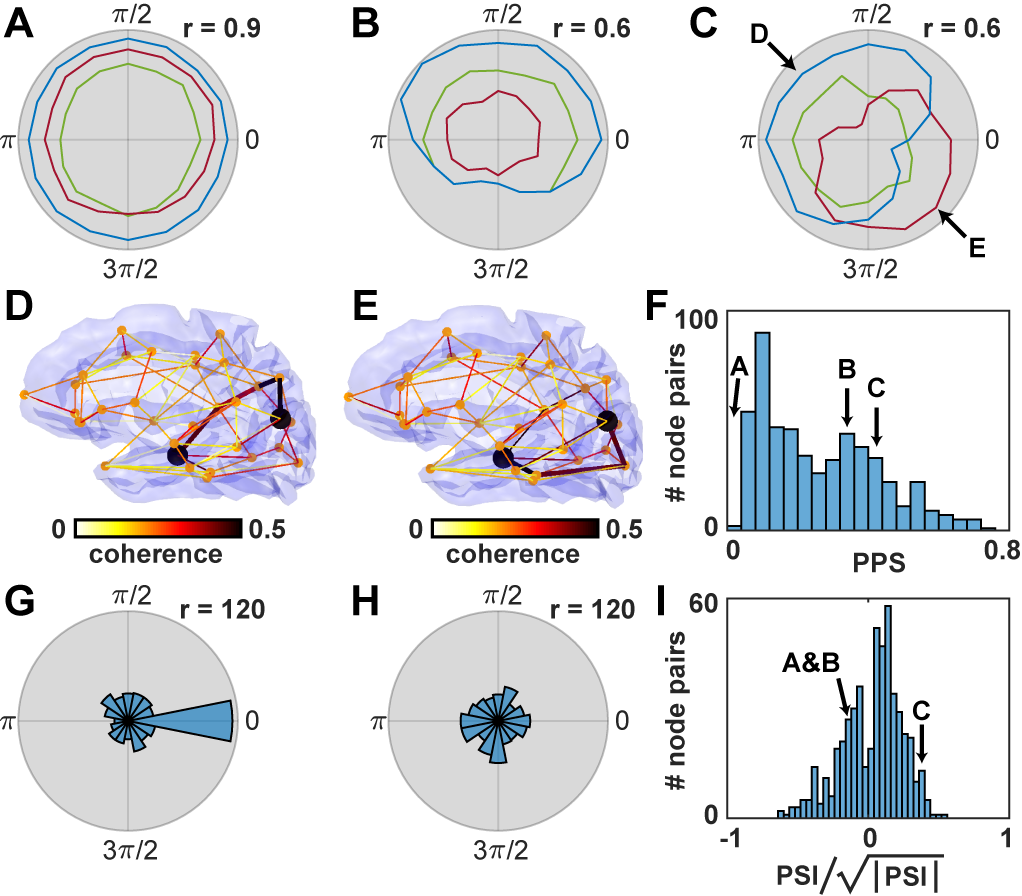

Supplement: S3 Fig — The panels correspond to Fig 7 in the main text. Please find the detailed panel descriptions there. (TIF) [file pcbi.1007551.s003.tif]
